# Stakeholders’ Perceptions of Benefits of and Barriers to Using Video-Observed Treatment for Monitoring Patients With Tuberculosis in Uganda: Exploratory Qualitative Study

**DOI:** 10.2196/27131

**Published:** 2021-10-27

**Authors:** Juliet Nabbuye Sekandi, Vicent Kasiita, Nicole Amara Onuoha, Sarah Zalwango, Damalie Nakkonde, David Kaawa-Mafigiri, Julius Turinawe, Robert Kakaire, Paula Davis-Olwell, Lynn Atuyambe, Esther Buregyeya

**Affiliations:** 1 Department of Epidemiology and Biostatistics College of Public Health University of Georgia Athens, GA United States; 2 Global Health Institute College of Public Health University of Georgia Athens, GA United States; 3 Infectious Disease Institute Kampala Uganda; 4 School of Public Health Makerere University Kampala Uganda; 5 Kampala Capital City Authority Kampala Uganda; 6 School of Social Sciences and Social Work Makerere University Kampala Uganda

**Keywords:** tuberculosis, adherence, mHealth, video directly observed therapy, Uganda, mobile phone

## Abstract

**Background:**

*Nonadherence* to treatment remains a barrier to tuberculosis (TB) control. Directly observed therapy (DOT) is the standard for monitoring adherence to TB treatment worldwide, but its implementation is challenging, especially in resource-limited settings. DOT is labor-intensive and inconvenient to both patients and health care workers. Video DOT (VDOT) is a novel patient-centered alternative that uses mobile technology to *observe* patients taking medication remotely. However, the perceptions and acceptability of potential end users have not been evaluated in Africa.

**Objective:**

This study explores stakeholders’ acceptability of, as well as perceptions of potential benefits of and barriers to, using VDOT to inform a pilot study for monitoring patients with TB in urban Uganda.

**Methods:**

An exploratory, qualitative, cross-sectional study with an exit survey was conducted in Kampala, Uganda, from April to May 2018. We conducted 5 focus group discussions, each comprising 6 participants. Groups included patients with TB (n=2 groups; male and female), health care providers (n=1), caregivers (n=1), and community DOT volunteer workers (n=1). The questions that captured perceived benefits and barriers were guided by domains adopted from the Technology Acceptance Model. These included perceived usefulness, ease of use, and intent to use technology. Eligible participants were aged ≥18 years and provided written informed consent. For patients with TB, we included only those who had completed at least 2 months of treatment to minimize the likelihood of infection. A purposive sample of patients, caregivers, health care providers, and community DOT workers was recruited at 4 TB clinics in Kampala. Trained interviewers conducted unstructured interviews that were audio-recorded, transcribed, and analyzed using inductive content analysis to generate emerging themes.

**Results:**

The average age of participants was 34.5 (SD 10.7) years. VDOT was acceptable to most participants on a scale of 1 to 10. Of the participants, 70% (21/30) perceived it as highly acceptable, with scores ≥8, whereas 30% (9/30) scored between 5 and 7. Emergent themes on perceived benefits of VDOT were facilitation of easy adherence monitoring, timely follow-up on missed doses, patient-provider communication, and saving time and money because of minimal travel to meet in person. Perceived barriers included limited technology usability skills, inadequate cellular connectivity, internet access, availability of electricity, cost of the smartphone, and use of the internet. Some female patients raised concerns about the disruption of their domestic work routines to record videos. The impact of VDOT on privacy and confidentiality emerged as both a perceived benefit and barrier.

**Conclusions:**

VDOT was acceptable and perceived as beneficial by most study participants, despite potential technical and cost barriers. Mixed perceptions emerged about the impact of VDOT on privacy and confidentiality. Future efforts should focus on training users, ensuring adequate technical infrastructure, assuring privacy, and performing comparative cost analyses in the local context.

## Introduction

*Nonadherence* to treatment of tuberculosis (TB) remains a significant challenge to meeting the World Health Organization’s End TB Strategy to reduce deaths by 90% and incidence by 80% [1]. Approximately 10 million new TB cases occur annually, and 1.5 million die from the disease worldwide [2]. An estimated 33% to 50% of patients who start treatment are nonadherent to their prescribed medication regimens, particularly in low- and middle-income countries (LMICs) [3]. Nonadherence to medication can result in the emergence of drug resistance, prolonged infectiousness, treatment failure, and relapse [4-6]. Poor adherence is particularly common in LMICs, where TB rates are high and resources for health care delivery are limited [7].

The Uganda National Tuberculosis Prevalence Survey reported a high TB case rate of 253 per 100,000 people in 2015 [8]. The Uganda National TB Program uses the recommended standard directly observed therapy (DOT) but implements a mixture of facility-based DOT and community-based DOT (CB-DOT) [9]. In practice, CB-DOT is predominantly used in urban settings such as Kampala city. CB-DOT typically involves a trained community DOT volunteer designated by the TB program or a treatment supporter (a family member, friend, or neighbor) as selected by the patient who is responsible for watching the daily intake of each medication dose [5,10,11]. Previous studies conducted in Uganda have shown the effectiveness of CB-DOT in rural settings, but mixed findings have been reported in urban settings [10,12,13]. Proper implementation and sustainability of standard DOT has been limited because of a lack of funding and its heavy reliance on volunteers [14]. Other factors such as a severe shortage of health workers, high cost of transportation, and the inconvenience of the need for face-to-face provider-patient contact largely impede the feasibility of DOT [7,12,13]. In the end, many patients take medications on their own and then self-report pill ingestion. The lack of an objective method to validate self-reported adherence highlights the need to explore alternative methods of medication monitoring.

The updated guidelines for the treatment of drug-susceptible TB issued by the World Health Organization in 2017 recommended the use of digital adherence technologies such as video DOT (VDOT) as an alternative method to monitor adherence [15]. Digital technologies have been shown to overcome the common systemic barriers to TB treatment delivery [16-19]. The VDOT process involves using a smartphone app to record and send daily medication intake videos to a secure computer system. The submitted videos are then accessed by health care providers treating TB for remote observation [20]. Although VDOT has been generally shown to be feasible, acceptable, and effective when evaluated in high-income countries and LMICs [20-25], low acceptability has also been reported among some users in LMICs [24]. In Uganda, a growing body of evidence on the acceptability of digital adherence monitoring abounds mostly in HIV-infected populations on treatment [26-29] but is limited in populations with TB [30]. Moreover, there are no published studies on the acceptability and perceptions of patients with TB and other stakeholders related to the use of VDOT in Uganda. This exploratory qualitative study was conducted to inform a pilot feasibility study of VDOT for adherence monitoring and support in Uganda. The findings in this paper add to the limited evidence needed to inform future implementation and scale-up of digital adherence technologies in Uganda and other LMICs.

## Methods

### Ethical Review

All participants provided written informed consent (including permission to audio-record the sessions for transcription and coding purposes) in English or Luganda as they preferred and were informed of their freedom to withdraw at any time during the interview. The institutional review boards approved the study at the University of Georgia, Office of Research (STUDY00004974), and Makerere University Higher Degrees, Research and Ethics Committee, in Uganda (Protocol #562). All participants were offered an equivalent of US $5.00 in Uganda Shillings at the end of the interviews as a refund for travel expenses and compensation for their time.

### Theoretical Framework

There is considerable evidence suggesting that theories of behavior change are useful for informing the effective design, uptake, and adoption of new health interventions by health care workers and patients for the management of TB [31]. Generally, before changes in behavior occur, intended users have to perceive it as valuable to their lives and accept the intervention. We adopted some constructs from the Technology Acceptance Model, which is an information systems framework for understanding how users accept and eventually use new technology [32]. The model posits that attitudes, perceived usefulness, and ease of use of technology predict the intention to use the technology, which subsequently correlates with actual use [32]. The strength of the Technology Acceptance Model is that it can be used to explore views from a broad spectrum of users, including patients and health care providers.

### Setting and Population

The study was conducted in Kampala, the capital city of Uganda, from April to May 2018. This work was accomplished through collaborative efforts between researchers at the Makerere University School of Public Health, the University of Georgia, and the Uganda National TB Program staff. Kampala district has the highest TB burden in Uganda, with nearly 25% of annually reported TB cases occurring in the metropolitan area of the capital city [9]. In Kampala, care for patients with active TB is delivered through public and designated private clinics supervised by the Kampala Capital City Authority, with an oversight from the Uganda National TB Program. Diagnosis and treatment services for TB are provided free of charge to all patients.

### Study Design

We conducted a cross-sectional qualitative study of 5 focus group discussions involving a total of 30 participants. The focus groups were composed of a minimum of 6 participants, as recommended for qualitative studies [33,34]. Two groups comprised patients with TB stratified by sex, 1 group had caregivers, 1 group had TB health care providers (nurses and clinicians), and 1 group had trained DOT community volunteer workers. The caregiver category was broadly defined as any person (a spouse, other family member, or a friend) who regularly provided supportive care, such as accompanying the patient during the TB clinic visits. A DOT community worker was a volunteer who was previously trained by the National TB Program and designated to support DOT in the community for patients attending a specific TB clinic. The patient focus groups were stratified by sex to create more homogeneous groups to foster an environment for free discussion. Owing to cultural norms in the Ugandan settings, men tend to dominate conversations and women tend to be less inclined to speak in the presence of men. Therefore, sex stratification was important whenever possible, and the focus groups of health care providers and DOT community workers were not stratified by sex, as the job positions and number of workers were somewhat fixed. We used a hypothetical scenario to describe the step-by-step process of VDOT, as none of the participants had prior experience with it. We then gathered participants’ perceptions of the potential benefits and barriers.

### Detailed Description of VDOT

**Figure 1:**
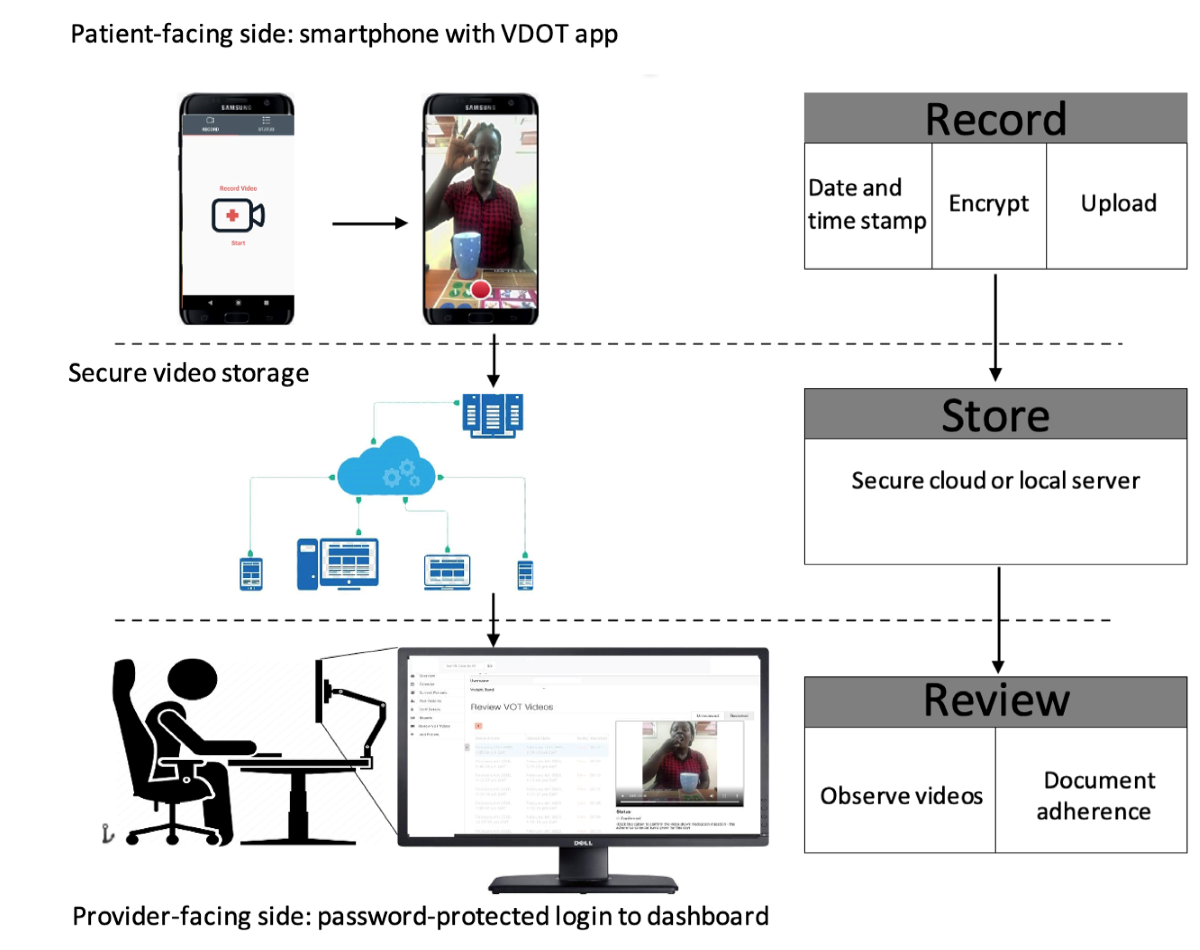
Schematic of the asynchronous video directly observed therapy system for monitoring tuberculosis treatment.

The hypothetical scenario involved the following: a description coupled with a demonstration of how VDOT works were offered to the participants before the start of interviews in the focus group discussions. The VDOT system, as shown in Figure 1, has 3 main components: a patient-facing side, which is composed of a smartphone app for recording and submitting videos; a secure Health Insurance Portability and Accountability Act–compliant cloud server that stores the encrypted videos; and a provider-facing, computer-based log-in system with a dashboard where submitted medication videos are watched and adherence is confirmed. Daily dosing can be monitored, and individual or aggregated reports can be generated within the system. The system also sends automatic text reminders to patients’ phone numbers. A second reminder was sent 8 hours later if the medication video was not received in the system. An example of these messages is “It’s time to take your pills and send a video. Taking your pills will help you to get cured.” A translated version of the message is also sent in a local dialect (Luganda), which is predominantly used in the Kampala region.

### Participant Recruitment and Enrollment

A purposive sample of adult male and female patients with TB with their caregivers was invited to participate in the focus groups. Participants were approached face to face with the help of a clinic nurse at the TB clinic under the National TB Program. Patients with TB were eligible if they were aged ≥18 years and were receiving treatment for at least 2 months under the usual in-person DOT to ensure that they were no longer infectious. Consenting participants were recruited from 4 public TB clinics. Community DOT volunteers were also selected according to the participating TB clinics to which they were attached. All the participants who were invited agreed to participate in the study.

### Data Collection and Focus Group Procedures

Focus group discussions were conducted in Luganda (a commonly spoken local dialect) for patients, caregivers, and community DOT workers and in English for the health care providers. The moderator (RT), a male qualitative interview expert, reviewed the purpose of the study and the agenda for the meeting. He then described the VDOT process and demonstrated how it works using a smartphone and the app. Participants were shown the special features of the app, password access to the app to ensure privacy, and how the videos were recorded and sent to the cloud system. In addition, the provider-facing dashboard and what happens during a video review session were shown on the computer. RT introduced the interview topics and posed questions, followed by probing. The assistant moderator (RK), a female social worker with vast experience in qualitative data collection, asked follow-up questions, audio-recorded the discussion, and captured nonverbal expressions to enrich the collected data. Interview guides were pretested and revised, and the final versions were used for the data collection process (see an example of the guide in Multimedia Appendix 1). RK and other trained research team members also administered a brief exit survey on demographics, phone ownership and use, and perceived acceptability rating. Focus group discussion sessions were conducted at a selected clinic over the weekends to minimize disruption of routine health care activities and ensure that nonparticipants were not present. Each session lasted between 75 and 125 minutes.

### Qualitative Data Processing and Analysis

To ensure data integrity, RT transcribed the data verbatim from the audio-recordings in an iterative process within 48 hours of completing the interviews. During transcription, RK reviewed the transcripts for accuracy by playing back the audio-recordings while reading the transcripts. In case of a discrepancy, the transcripts were edited to match the audio-recordings. The transcripts were then imported into ATLAS.ti for thematic content analysis using an inductive approach. Two authors (RT and RK) independently analyzed 3 phases: data immersion, coding, and coding sort. In the first phase, the research team led by JS and EB reviewed the transcripts several times to achieve familiarity and identify emerging issues. In the second phase, the authors flagged relevant transcripts with appropriate descriptive words (codes). In the third phase, the reviewers met and harmonized the independent codes. The harmonized codes were then combined to form categories, and the categories were combined to form emergent themes and subthemes. A third independent reviewer (VK) with vast qualitative analysis expertise verified the codes and themes. The reporting of the study results was guided by the Consolidated Criteria for Reporting Qualitative Studies guidelines [35]. Data from the exit survey were summarized as frequencies, means, SDs, and percentages. Summary statistics of the acceptability rating scores on a scale of 1 to 10 are presented as means and SDs (Table 1). For this study, the scores were further categorized into 3 groups to reflect levels of acceptability as low (1-4), moderate (5-7), or high (8-10).

**Table 1:** Baseline characteristics and technology experience of focus group participants in Kampala, Uganda, 2018 (N=30).

Demographic characteristics		Stakeholder category					
		Female patients with TB^a^ (n=6)	Male patients with TB (n=6)	Health care providers treating TB (n=6)	DOT^b^ community workers (n=6)	Caregivers (n=6)	All participants^c^
Age, mean (range)		28.7 (20-37)	29 (24-35)	39.3 (30-57)	34.7 (25-62)	40.7 (28-54)	34.5 (10.7; 20-62)
**Highest level of education, n**							
	Primary	3	3	0	2	0	8 (27)
	Secondary	3	1	0	0	2	6 (20)
	Tertiary or university	0	2	6	4	4	16 (53)
**Cell phone ownership, n**							
	Basic feature phone only	4	3	0	2	3	12 (40)
	Smartphone only	2	2	4	3	3	14 (47)
	Both regular and smartphone	0	1	2	1	0	4 (13)
**Cell phone experience, n**							
	Uses cell phone camera regularly—yes	2	3	6	4	4	19 (63)
	Takes selfies—yes	1	2	4	3	3	13 (43)
	Uses phone to take videos—yes	1	3	5	5	4	18 (60)
	Sends photographs or videos via phone—yes	1	4	6	5	4	20 (67)
	Uses phone for internet access regularly—yes	1	4	6	4	4	19 (63)
	Uses WhatsApp or Facebook on phone—yes	1	5	6	5	5	22 (73)
**Level of perceived acceptability for VDOT^d^ on a scale of 1 to 10, n**							
	Value, mean (range)	N/A^e^	N/A	N/A	N/A	N/A	8.23 (1.87; 5-10)
	1-4 (low)	0	0	0	0	0	0 (0)
	5-7 (moderate)	1	2	2	3	1	9 (30)
	8-10 (high)	5	4	4	3	5	21 (70)

^a^TB: tuberculosis.

^b^DOT: directly observed therapy.

^c^Values in the column *All participants* are presented as mean (SD; range) for mean age, and as n (%) for the remaining characteristics.

^d^VDOT: video directly observed therapy.

^e^N/A: not applicable.

## Results

### Characteristics of Participants

A total of 5 focus groups were conducted with a total of 30 participants, with an average age of 34.5 (SD 10.7) years and an age range of 20 to 62 years. All participants owned a cell phone, but more health care providers owned smartphones compared with other categories. Overall, experience with cell phone use was modest, with at least 60% (18/30) using their phones to take photographs, record videos, or use the internet. However, patients reported relatively lower use of these phone features of interest compared with health care providers. Participants expressed mixed views about acceptability, with ratings varying somewhat across categories. The mean score for acceptability was 8.2 (SD 1.87), with a range of 5 to 10 on a scale of 1 to 10. About 70% (21/30) of participants rated VDOT as high, with a score of >8, whereas 30% (9/30) perceived it as moderately acceptable. Detailed information on the baseline characteristics of the participants is provided in Table 1.

### Overview of Results

The results from the focus group discussions are broadly categorized into perceived benefits and barriers. Within each category, we present themes and subthemes that emerged across the 4 categories of participants, including patients, caregivers, community DOT workers, and health care providers.

### Perceived Benefits of VDOT

Three themes and interrelated subthemes emerged from discussions on perceived benefits. These included easy monitoring and support of adherence, enhancing patient-provider communication, and saving time and money. The subthemes presented under ease of monitoring reflect an aspect of this theme. These perceived benefits of using technology for monitoring TB medication adherence could indicate the degree of acceptability of VDOT.

#### Easy Monitoring of Medication Adherence

Participants generally perceived that using VDOT would make it easier to monitor medication adherence. Community DOT workers and TB health care providers revealed that sometimes patients may falsely report taking their medications under the standard *DOT* method. For context, the TB health care providers in the focus group acknowledged that the Uganda TB program faces practical challenges, such as limited personnel, which makes it difficult to administer proper in-person DOT. Ideally, DOT should involve consistent observation of the patient’s daily swallowing of medications by a health worker. However, in practice, most patients are seldom supported by designated community DOT volunteer workers.

##### Subtheme A: Objective Evidence of Adherence

Patients often take medicine on their own (self-administered treatment) and then self-report adherence at monthly clinic visits. In such situations, the health workers believed that VDOT would be more reliable because it provides video evidence of swallowing the medicines instead of relying on patients’ self-reports:

I think VDOT is more evidence-based, you find that you’ll be seeing what the patient is doing not like DOT where you wait for the patients to tell you what they did.

TB health provider #2

But this video will be able to show you that this person has taken drugs, because you have to place the pills on the tongue to show us that you have really taken drugs and take water, then you put a second pill and take some water...

Community DOT worker #5

##### Subtheme B: Timely Follow-up of Missed Doses

Patients perceived VDOT to be beneficial for the early identification of missed doses and follow-up to solve problems. Patients thought the technology would help a health worker to find out what is going on with a patient before the next visit and also plan to intervene early if a patient is not doing well:

When using video, it helps the health worker to follow you up, it becomes easy for the health worker to follow you up like yesterday you were not able to take your drugs then he can even remind you that your time has passed and you should have taken your drugs. At times, the health workers think we don’t take the drugs.

Patient #5, female

The health worker will be able to follow up the patient and she will be able to know how you are doing at times the health workers think that we don’t take the drugs, they know some of us don’t take the drugs and when I tell him or her that’s what he takes. But on the video [VDOT] the health worker will be on the right track.

Patient #4, female

##### Subtheme C: VDOT Is Convenient and Efficient

Participants described VDOT as a convenient and efficient strategy for observing many patients within a limited time while being seated at one place compared with a community DOT worker who moves from place to place to observe medication intake:

I think the VDOT may be more effective like p#4 [another participant] said that you may be having 50 patients to attend to or follow up but I am not going to be around to watch all these people when they are taking their drugs in one day,...[with VDOT] you will sit on your computer in one place and monitor all the people and you will be able to follow up people by calling only those whose videos are missing...

Community DOT worker #3

You know we left analogue [“old fashioned ways”] and we are now in digital [“modern ways”], and everything is digitalized so this method seems to be more improved than the analogue method where you have to monitor someone by visiting that person, this one will help the health workers to easily monitor a patient when the patient is in his/her room and the health worker is in his office without having to wait for the patient to come...

Caregiver #5

#### Improved Patient-Provider Communication

Although VDOT would take away the in-person contact between patients and health workers provided by DOT, some participants believed that VDOT would increase provider-patient communication. Patients were pleased to learn that the asynchronous VDOT sessions would have no time limitations, instead these sessions would allow them to share their treatment-related problems. Health workers agreed that by eliminating the time constraints, VDOT would enable them to focus more on patients’ needs and concerns, thereby fostering good patient-provider relationships and improving medication adherence:

[Patient] talking when taking drugs creates a good atmosphere between the patient and the health worker. With time, the patient will develop trust in the health worker because of that routine interaction. This will encourage him or her to continue taking medications.

TB health care provider #3

This video is going to help the patient to air out his/her complaints, not complaints but how the patient is progressing with the treatment, or the side effects the patient is getting out of these drugs like my legs paralyzes, I vomit, I feel pain in such and such a place, so this person will have got a platform to air out his or her problems early than waiting to see the health worker [at the clinic or home visit]...

Community DOT worker #6

#### VDOT Saves Money and Time

Both health workers and patients believed that VDOT could potentially save both time and money. Health workers pointed out that patients’ visits at home and workplaces for DOT are costly, especially when visiting multiple patients daily. They thought that the cost of internet data might be much lower than the transportation costs. Participants also believed that time spent in transit to patients’ homes and workplaces, especially with traffic jams, could be used in ways that would be more beneficial to the patients:

It [VDOT] is straightforward; everywhere you are, any time you want. [However] this health worker who has to come to you may lack time, she/he may have other things to do. Also [with VDOT], the health worker will save on transport. Where she has used UGX 5000 [on transportation], she will use UGX 400 on the cost of internet data.

Patient #4, female

With a video in practice, I will not have to waste my time visiting many patients or even telling the DOT volunteers to schedule visits with many patients. We shall only visit those who do not send their videos and use time saved to do other work like the paper work at facilities.

TB health care provider #1

### Perceived Barriers to VDOT

Although VDOT was generally perceived to have several benefits by the respondents, they highlighted some potential barriers to its use. A total of 4 themes and related subthemes emerged prominently from the discussion. These included concerns about limited technology skills among users, inadequate technical infrastructure to support VDOT use, costs related to using the technology, and disruption of domestic routines for female patients. Subthemes that reflect aspects of inadequate infrastructure are also presented. Perceived barriers to VDOT are likely to reduce the acceptability and speed of adoption during large-scale implementation. The detailed findings and related quotes are provided later.

#### Limited Technology Usability Skills

Participants were concerned that patients with limited technological skills would have a difficult time using VDOT successfully. Although all of the participants owned cell phones, very few patients owned smartphones and were not very familiar with features such as video functions or mobile apps. Several participants described the difficulties they faced while trying to operate a smartphone:

Because of my age, I might have challenges in taking a good video, because I cannot operate my phone [smartphone] very well or I cannot focus it [the camera] very well so what I do I just go to someone to ask that person to help me to do what I want to do with my phone, so smart phones are complicated for some people...

TB health care provider #4

The challenge I see is that like my brother here P5 [fellow participant in group] who said that he doesn’t know how to use a smart phone, he will find it hard to record a video and send it even when he is given MBs.

Patient #4, male

#### Inadequate Technical Infrastructure

##### Subtheme A: Poor Cellular Network Connectivity and Internet Access

Some community DOT workers and health care providers expressed concerns about the unstable cellular network coverage in some areas. They worried about the disruption of the video recording, submission process, and review by the health workers. They also stated that an unstable network could result in more internet data being used up, thereby increasing the operational costs of using VDOT:

If there is no network or internet, you [a health provider] will not be able to receive that video...If there is no network where this person [patient] is, he [patient] will not be able to send the video, and you [health provider] will not get the video that day, or the following day.

Community DOT worker #2

Some areas have poor internet network, and at times there is no network at all...because of poor network, where you would have used five hundred shillings worth of internet M.B.s, you find yourself rather using two thousand shillings worth.

Community DOT worker #6

##### Subtheme B: Unstable Electricity

Some participants also had concerns about unstable electricity supply that might prevent some patients with TB from charging the smartphone battery, interrupt daily video recordings and uploads, and disrupt adherence monitoring:

The challenge...[is] power may go off when you have not charged the phone and yet it is time to take the drugs, power is off, but you want the health worker to see you so you have nothing to do.

Patient #2, female

Sometimes we run out of battery power, then you have a power blackout even going on for two days, and the phone is off. So, I think that could be one of the challenges...the phone is not charged...also without power you do not have enough light to take your video.

TB health care provider #2

#### Recurrent Costs Associated With Using VDOT

Participants in each group expressed concerns about the costs associated with using VDOT. Most patients stated that although they would be willing to record videos of themselves while taking medications, the recurrent costs of accessing the internet would be a major barrier. There were concerns that for some low-income patients, daily recording of videos might require them to choose between purchasing food and internet bundles:

There is a situation when it is time for taking drugs, and you have to take a video. At that moment you may be having only 500UGX on your table. You will not use that money to buy internet M.B.s when you don’t have money to buy something to eat...so you will find yourself missing one of the two.

Patient #5, male

With the data everyone is suffering with this infection differently at times you have no strength and you can’t afford to go and work so you find that even data will be another challenge because at times I take a week without working when I feel so weak and it comes to 8.00am when I don’t want to get out of the bed.

Patient #1, female

#### VDOT May Disrupt Domestic Work Routines

Some female participants were concerned about how VDOT might interfere with their domestic routines, for example, cleaning, cooking, and caring for the family. These duties are mostly performed by women in the Ugandan context, and they often fill up the entire day:

In the morning, we usually have no time. You are preparing yourself or preparing children for school. Now you are telling me that I [should] get the phone [to record a video]? The time is little.

Patient #3, female

### VDOT as a Potential Benefit and Barrier

Mixed perceptions of confidentiality, privacy, and other closely related issues emerged prominently in the discussions across all participant categories. Although some respondents believed that VDOT could potentially enhance privacy, others expressed fear that their privacy and confidentiality could be breached. Both ends of the spectrum are presented as subthemes to show the perceived benefits of and threats to privacy or confidentiality.

#### Subtheme A: Benefits of Preserving Privacy

Participants believed that the VDOT method would allow patients to take their medications discreetly, unlike DOT where community members witness health workers visiting their neighbors daily for medication intake. Patients revealed that they would be motivated to use VDOT if their TB status would be kept private, and videos would not be witnessed by non–health care staff:

I don’t want health workers at my home; everyone will know that I have TB, which will scare me from taking my drugs. So, the big thing VDOT will save us [from] is the issue of health workers following us in the community; there is no privacy because you will come with a car which has the hospital words, it will expose me, so for this one [VDOT] I will be in my room and I will take a video and send it to the health worker and that will be all.

Patient #3, male

With the DOT method, they [DOT workers] come to my home. If they do not find you, they will ask a neighbor where you work. So you will just see them coming to your work. Or they will find you in the market and people will be wondering why they are looking for you...but with VDOT you will do your things in a private way.

Caregiver #4

#### Subtheme B: Perceived Threats to Privacy

Unintended disclosure of the TB disease status to close family members or workmates was cited as one of the concerns that could result from the use of VDOT. Some participants feared that videos sent to a health worker could be shared or accessed by other people, and others worried that the process of recording a video might attract unwanted attention from a passerby in the vicinity. Some patients also expressed perceptions of stigma as a result of unintended disclosure of the TB disease status:

The video we would use it but now like us who are married I had already talked about it, I am not married fully because my husband comes from the other end he has another home [polygamous situation] I have never disclosed to him that I have TB he may get that video there he gets to know and its bad.

Patient #2, female

My fear is taking a video and it is shared with someone else...when people realize that you have TB they try to dissociate from you. People are scared of TB, even me. I also protect myself with the people I work with, so it does not require people to know that you have TB.

Patient #1, male

#### Subtheme C: Breach in Confidentiality and Security of Videos

Patients, health workers, and community workers expressed concerns and fears about the confidentiality of information in the videos once received on the health system side. Multiple participants asked about who would have access to the videos and whether the health workers could be trusted to protect the videos. Some patients feared that uploaded videos might be shared on social media sites or be used for advertisements without their permission:

I would be concerned about that because in most cases people misuse social media, so I may send you my video taking medications, and the video may be misused and sent for advertisement or something like that.

Patient #5, male

#### Subtheme D: Lack of a Private Place to Record Videos

Some participants raised another threat to privacy stemming from the lack of a convenient place to record videos. They noted that some patients might be uncomfortable or embarrassed to reveal their living or work environment to health care providers through video recordings. This may deter patients from recording and uploading VDOT videos:

People will even fear to record videos in such environment [their homes]...they don’t want you [the health worker] to know where they stay.

Community DOT worker #1

It would be difficult for our clients because most of them work in the market, [or in] taxis where there so many people. So getting a private place to take his medicine and taking the video will be very difficult.

TB health care provider #2

When you are at [the] work place, there are many people. As you know the setup of the town, we are many people working from [a] shed. For you [a patient] to get out the [TB] drugs then [record] the video because you are sending it to your health care worker, it would not be good.

Patient #5, female

## Discussion

### Principal Findings

In this study, we explored perceived acceptability and benefits of and barriers to using VDOT among patients with TB, their caregivers, health care providers, and community DOT volunteer workers in Uganda. Although we used a hypothetical scenario of VDOT to interview participants who had no prior experience, we gathered very rich information about perceived benefits and barriers. We found that VDOT was perceived as acceptable by most participants, despite some differences between patients and providers. Prominent benefits related to the ease of patient monitoring; timely follow-up of missed doses; and enhancement of provider-patient communication, efficiency, and cost savings. The perceived barriers cited were limited technology skills; suboptimal technical infrastructure; costs of smartphones; internet access; and disruption of routine domestic work, especially for female patients. There were mixed perceptions about the impact of VDOT on privacy and confidentiality, particularly with unintended disease closure, potentially resulting in stigma. Similar perceptions of structural and privacy- and patient-related barriers to VDOT implementation have been previously reported [24,36,37]. For example, some studies have reported that video recordings and repeated text reminders are perceived as being overly intrusive [37,38]. In contrast, a study in India reported that patients with TB perceived VDOT as being more private than DOT [24]. The mixed findings underscore the need for a critical evaluation of potential barriers within local contexts. To our knowledge, this is the first study to examine the perspectives of key stakeholders in the use of VDOT in Uganda. More studies are needed to contribute to the sparse evidence base on the topic.

The perceived benefits of VDOT highlight aspects that can be enhanced to increase acceptability among users. The ease of monitoring, convenience, access to objective evidence of dosing, and facilitation of timely follow-up have also been reported in previous studies [23,25,37,39]. Health care providers and patients perceived VDOT as a cost- and time-saving approach in the long run compared with costs that are likely to be incurred with frequent travel when using in-person DOT. Similar findings were reported in a study of health care providers in the private sector in urban Vietnam [39]. In addition, 4 studies quantified the time spent on the treatment observation process and found a significantly larger saving in time and money compared with the usual DOT [21,36,40,41]. VDOT was also perceived to enhance health care provider–patient communication, which in turn affects patient engagement with their treatment [42]. The consistency in these positive aspects makes VDOT a promising patient-centered approach for TB disease management.

The perceived barriers to using VDOT were mostly related to technology usability skills, given the limited experience with smartphones and apps. The technology usability barrier is likely to affect mostly older patients and those without formal education. Similar findings have been reported in VDOT studies conducted in Vietnam, Cambodia, and South India [23,37,39]. In our experience with the VDOT pilot study, intensive training with clear instructions to patients helped them gain the required skills. Inadequate network connectivity and electricity are structural barriers that are more challenging to overcome at the patient or programmatic level. Failure to charge a dead phone battery is one of the most common reasons for missing videos in the VDOT system [43]. The use of solar power banks for charging smartphones might be a short-term solution where electricity is unstable. The absolute costs of smartphones with internet data plans have also been documented as potential barriers in other studies [23,41]. The cost of smartphones on the global market is gradually decreasing, thereby boosting ownership, even in LMICs [44]. Our VDOT pilot study in Kampala showed that 70% of the participants owned a smartphone [43]. However, smartphones may remain unaffordable for some patients with TB. One possible way to minimize inequitable access to digital interventions is to set up a loaner system where patients can borrow and return their phones on completion of treatment. We used a similar system in our VDOT pilot study in Uganda, which worked well [43]. Regarding the cost of the internet, preloading the phones with prepaid internet could be a good option, but it raises other concerns of misusing it for other personal activities. Other creative ways can be explored to address the cost of internet access to support patients’ video submissions. For example, public-private partnerships between the National TB Program and telecommunication companies might tap into resources designated for corporate social responsibility from the business side. Gender roles emerged as a potential issue, especially for women, but have been rarely cited [38], warranting the need for more research. Despite these perceived barriers, patients and health workers believed that VDOT would be more flexible, convenient, and patient-friendly if the main barriers are addressed. Cost and cost-effectiveness studies are needed to inform the implementation and scale-up of VDOT [44].

Privacy and confidentiality issues with the use of VDOT were raised as perceived benefits and potential threats. Previous VDOT studies conducted in India, the United States, and Vietnam have also reported similar mixed concerns with privacy [24,25,39]. The importance of preserving confidentiality, guarding against unintended disease disclosure, and stigma also emerged as prominent themes in studies on acceptance of mobile health (mHealth) interventions among people living with HIV in rural and urban Uganda [26,30,45]. Indeed, the use of any mHealth interventions in monitoring treatment should be ethically comparable with the standard of care [38]. Specifically, for VDOT, the major privacy concerns raised have been addressed by several security features such as a unique personal identification number to facilitate secure log-in into the phone app, video encryption, secure cloud server, and password-protected log-in to the health system dashboard for the health care provider. However, to minimize feelings of mistrust, providers need to reassure patients that caution should be taken to prevent intentional breaches. It is also important to understand the complex cultural perspectives related to perceptions of privacy and cater to them accordingly. For example, some patients in South India perceived repeated adherence text message reminders as intrusive [37]. Finally, patients should also be encouraged to be active players in the process of protecting their own information, for example, by finding a private place to record videos and not sharing their personal identification numbers. DiStefano and Schmidt [38] proposed a valuable framework to guide ethical planning, implementation, and evaluation when using mHealth interventions. The collective goal is to minimize stigmatization and preserve patient autonomy [38].

Broadly, digital adherence technology studies on VDOT, electronic pillbox or Medication Event Reminder Monitor, and 99DOTS performed in LMICs have shown promising acceptability patterns among TB populations [22,37,43,46-48]. General lessons can be learned and applied across interventions and populations. For example, high acceptance in recent studies of VDOT was related to perceptions of convenience, ease of use, and perception of better privacy, whereas lower acceptance was mostly tied to technology skills, app glitches, and cellular connectivity challenges [23,24,36]. In India, a qualitative study evaluating differences in acceptability of 99DOTS, a low-cost, phone call–based strategy for reporting doses, found that high acceptance was related to improved patient-provider communication and the convenience of reduced clinic visits, among others [48]. Similar to our study, low acceptance was related to concerns about cell phone access, technology literacy, and poor cellular connectivity. Health care providers specifically expressed concerns about inadequate training in the use of the technology, changes in workload, and a lack of needed 99DOTS supplies. Although the health care providers in our VDOT study only had a hypothetical exposure, it is possible that similar challenges could arise. In a qualitative study using the electronic pillbox in patients with TB in Vietnam, the technology was perceived to be useful. However, the study participants pointed out that it would be most beneficial as a medication reminder for older patients. The device was less acceptable for people who worked outside of their homes, as they thought that the device was inconvenient to carry around [47]. Concerns about stigma, disease disclosure, or other privacy issues and costs related to technology seem to be crosscutting but to varying degrees from one study to another. Further research is required to expand our understanding on this key area.

### Strengths and Limitations

This study has several strengths and limitations that should be considered when interpreting the findings. To our knowledge, this exploratory qualitative study is among the first to document the perspectives of TB stakeholders in relation to VDOT in Uganda. It provided helpful insights into potential barriers and benefits that informed the design of the quantitative pilot study that subsequently followed [43]. One limitation of this study was that we used hypothetical scenarios of VDOT; therefore, respondents had no real-life experience using the technology intervention. This could have resulted in increased socially desirable responses and perhaps limited the ability of the respondents to envision potential facilitators and barriers comprehensively. Despite this limitation, respondents identified some critical issues such as cost of internet, the smartphone, and privacy or confidentiality concerns. The sample of respondents was limited to urban Uganda; therefore, findings may not necessarily be generalizable to all TB stakeholders in this local setting. The small number of focus group discussions could have fallen short of reaching the point of saturation. Overall, the findings of this study build on the sparse evidence on the acceptability of digital adherence technologies and their use in TB management in LMICs. More robust research is still needed to explore various aspects of acceptability, feasibility, and ethical issues related to VDOT. Other future research priorities have been highlighted as accuracy, clinical effectiveness, and cost-effectiveness of digital technologies [44].

### Future Implications

In the real world, successful implementation of VDOT will require both patients and health care providers to be equipped and willing to use the system. Users must have an adequate level of skills to operate the smartphone, mobile app, and technology system on the back end for health care providers. The baseline characteristics showed that the cell phone technology experience was higher among health care providers than among patients. This will require intensive training and perhaps run-in periods needed to allow users to gain a baseline functional level of skills at the beginning of a VDOT monitoring program. It is plausible that as individuals use VDOT, their level of comfort and usability skills will increase over time [49]. Special considerations must be made for subgroups such as older adults who may need more time to acquire new technology skills [50]. Any modifiable barriers must be addressed to ensure high uptake and sustained engagement with VDOT. Context- and culture-specific barriers for patients and health care providers must be evaluated before deploying the technology. The influence of digital technology use on gender roles emerged as a new aspect with little or no information from previous studies; therefore, further research is needed, especially in the African context.

### Conclusions

VDOT was relatively acceptable and perceived as beneficial by most study participants despite the potential technical and cost barriers. There were mixed perceptions about privacy and confidentiality issues related to the use of VDOT. Although some participants thought it would increase patients’ autonomy and privacy, others indicated fears about unintended disclosure of one’s disease status that could lead to stigma. Future efforts should focus on training users, ensuring adequate technical infrastructure, assurance of privacy, and comparative cost analysis studies in the local context.

## Appendix

Multimedia Appendix 1 Patient focus group interview guide.

## Abbreviations

CB-DOT: community-based directly observed therapy
DOT: directly observed therapy
LMIC: low- and middle-income country
mHealth: mobile health
TB: tuberculosis
VDOT: video directly observed therapy
