# Supplementary material for: Stakeholders’ Perceptions of Benefits of and Barriers to Using Video-Observed Treatment for Monitoring Patients With Tuberculosis in Uganda: Exploratory Qualitative Study
Source: JMIR Mhealth Uhealth. 2021 Oct 27;9(10):e27131. doi: 10.2196/27131 (PMC8581755; doi:10.2196/27131)
Supplement: Multimedia Appendix 1 [file mhealth_v9i10e27131_app1.docx]

**VDOT Pilot Project Focus Groups**

**Makerere University and University of Georgia (UGA)**

**PATIENT FOCUS GROUP FACILITATOR GUIDE**

| Introduction |
| --- |
| I appreciate your help with the consent forms and the brief survey. A few notes about our session today. This session will be recorded, but your name will not appear in any report or summary of the session. All of your comments are very important and we would like to give each person the opportunity to talk about their experiences or express their views. It is also important to remember that what is talked about here is confidential and that we respect everyone’s privacy and keep our conversation in this room.  Ok, now let’s get started…  People who work in health care know that it is important to be able to help TB patients take their medications. Directly observing patients taking their medications has been shown to significantly improve adherence to treatment, but we are aware that this can be time-consuming and inconvenient for patients. We want to understand better whether we can use cell phone technology to remotely watch patients taking their medications.  First, I will now take a few minutes to review the usual care known as Directly Observed Therapy (DOT) and then I’ll explain and demonstrate how VDOT works in real life. [The Focus Group moderator will show the participants how VDOT works using a flow diagram first then demonstrate in practice using a smart phone pre-loaded with the VDOT App, then the dashboard on the computer].  ***Moderator:*** *Be sure to highlight the need for a smart phone, cellular network and internet*. |
| **Mobile VDOT Procedures: Are they appropriate and useful?** |
| Let’s start by getting an idea of how familiar the members of our group are with using cell phones…  Q1. What experiences have you had using a cell phone to take a picture or video and send it to someone? Have you seen someone else do this?  Q2. If you were asked by the health work to use to receive a cell phone that can record video while taking your medicine to confirm that you swallowed your medicine, how would you feel? Probe: How useful would this VDOT system be? What problems do you think you or other patients might have with using cell phones to send a video?  Q3. How do you think such problems could be overcome?  Q4. In what situations do you think VDOT would not be good to use?  Q5. Will standard DOT or VDOT be more effective in helping patients to take all of their medications? Why? Why not?  Q6. If you were going to use the VDOT what would make it easier for you?  Q6.1. Let’s say that you were instructed to take your TB medication at 1:00 pm, how hard would it be for you to find a place to record this process and send it to the nurse (by “hard” I mean finding a private place, finding the time to do it, etc)  Q6.2 Now what do you think would make it easier for you to do this? |
| **Perceived benefits and barriers: VDOT Advantages/Disadvatages** |
| Q7. Let’s think about comparing the two kind of directly observed therapy, the standard way and then using cell phone video…In your opinion what advantages would VDOT have over standard DOT?  Q8. What disadvantages does VDOT have compared with standard DOT? |
| **Technical Advantages and Disadvantages of VDOT** |
| Q9. What advantages do you envision with using the cell phone technology to monitor patients on treatment? What would you like to see in place if you were going to try out this new process to send video information?  Q10. What barriers to do you think might hinder the use of the cell phone technology in monitoring TB patients on treatment?  Q11. Would it help patients remember to take their medication and send their video recordings if they received a reminder message on their phone everyday? |
| **Final Thoughts - End of the Session** |
| Q12. What other ideas do you think could help people use the cell phone for taking their TB meds? Perhaps something that we have not talked about yet, that may improve our ability to use this technology for medications? |
| **End of Session** |
| Conclusion: This concludes our focus group session. I very much appreciate your participation today. What we have learned from you is very important in order to know how to best implement the VCP-DOT Pilot program in our community.  **Once again, thanks.** |
